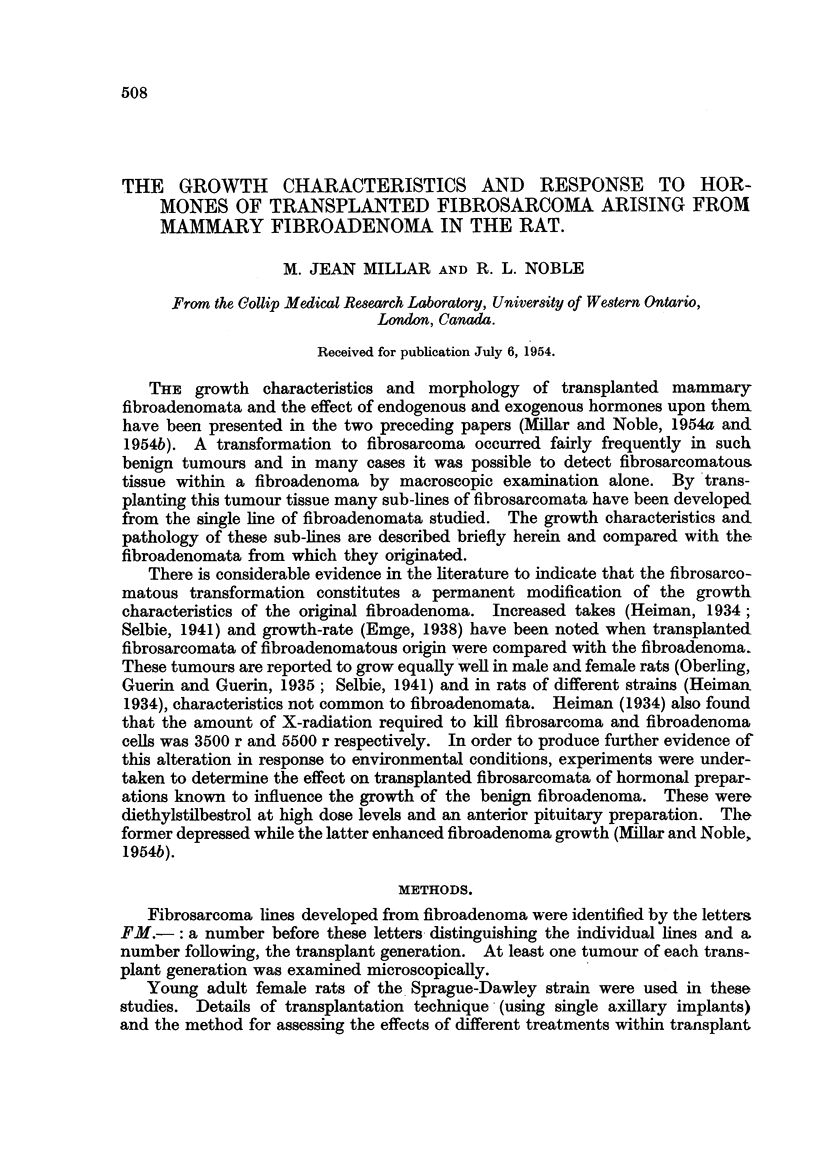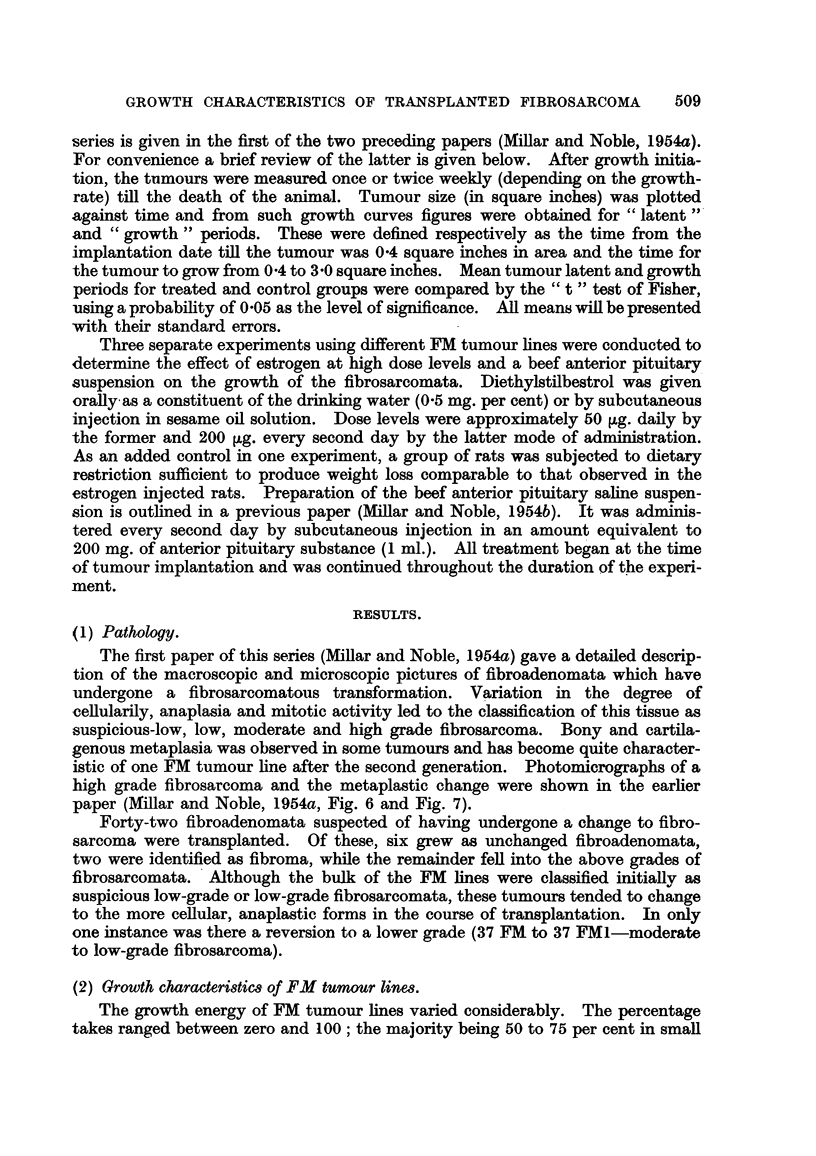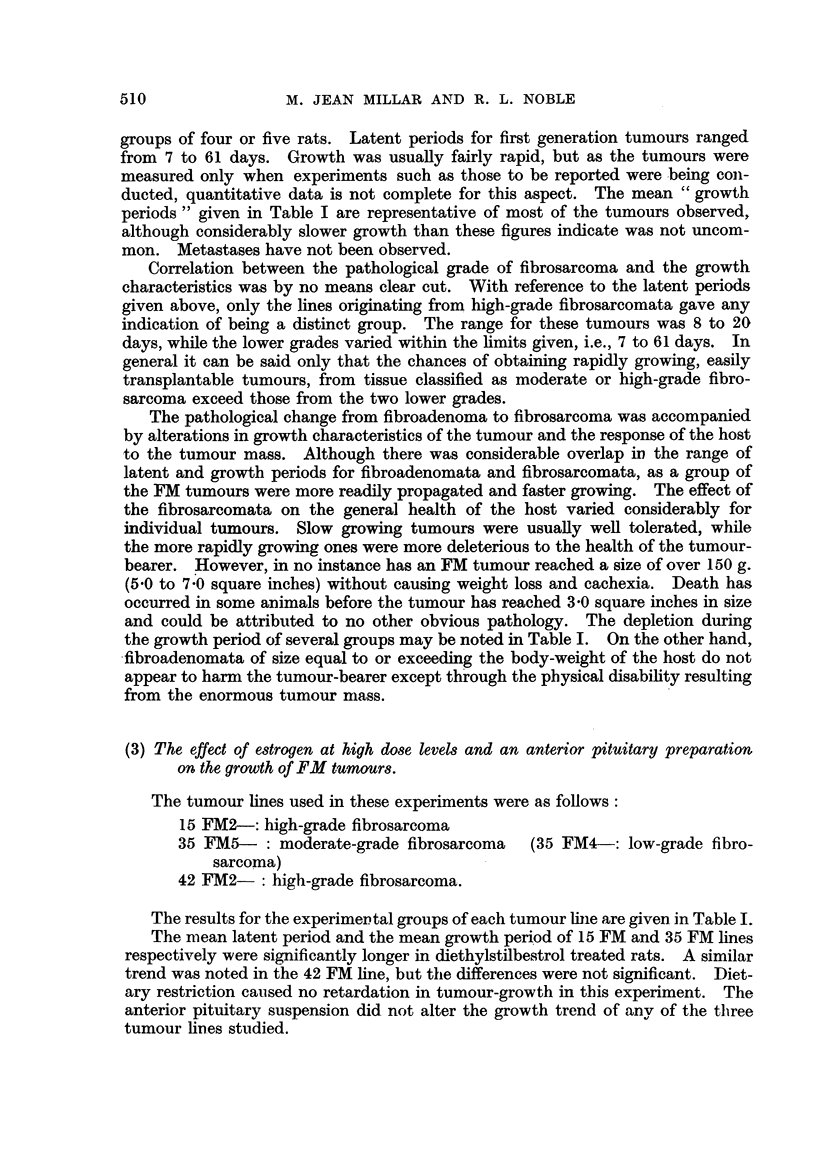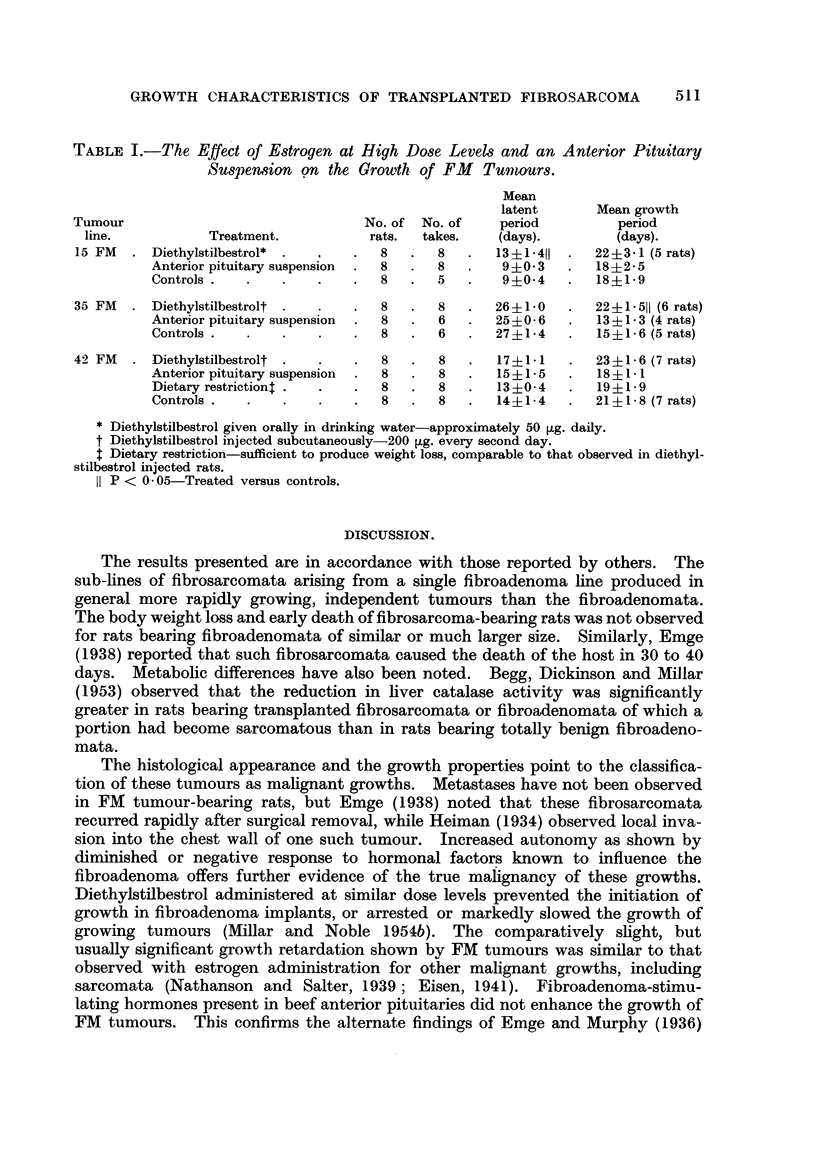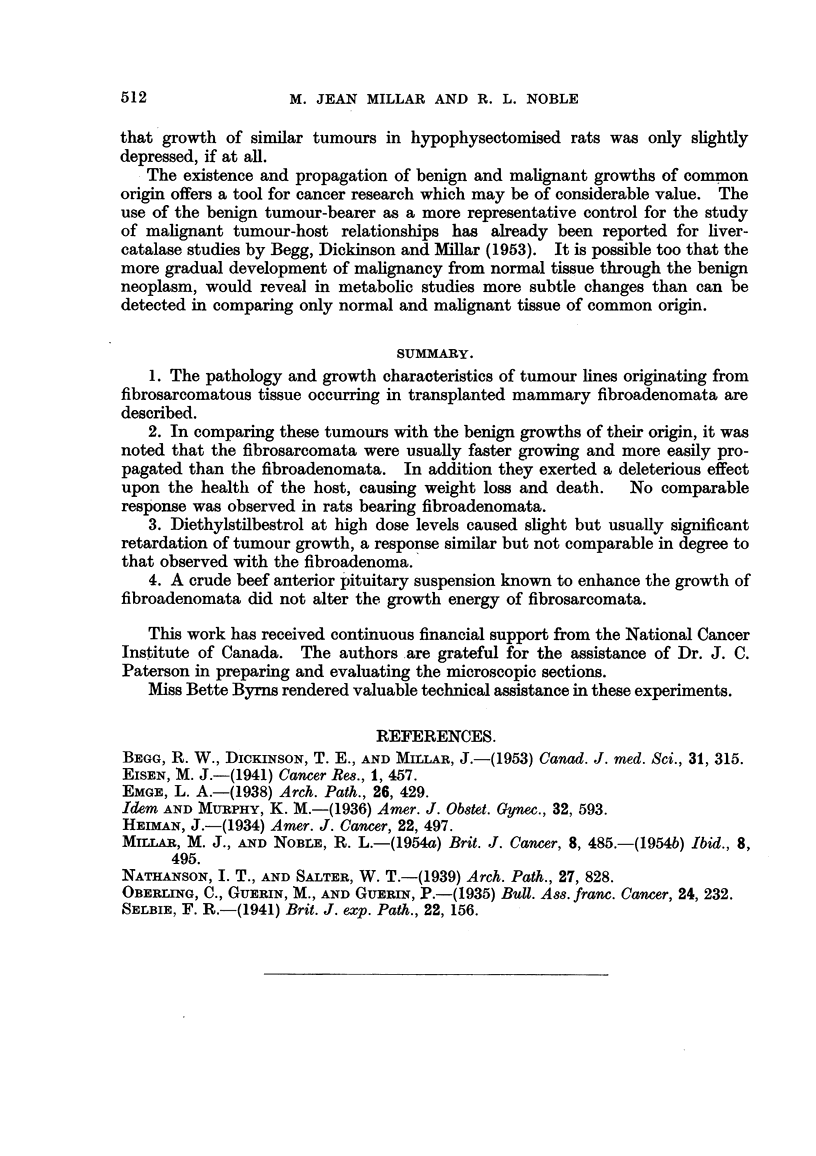# The Growth Characteristics and Response to Hormones of Transplanted Fibrosarcoma Arising from Mammary Fibroadenoma in the Rat

**DOI:** 10.1038/bjc.1954.53

**Published:** 1954-09

**Authors:** M. Jean Millar, R. L. Noble


### THE GROWTH CHARACTERISTICS AND RESPONSE TO HORMONES OF TRANSPLANTED FIBROSARCOMA ARISING FROM MAMMARY FIBROADENOMA IN THE RAT.

M. JEAN MILLAR AND R. L. NOBLE

*From the Collip Medical Research Laboratory, University of Western Ontario, London, Canada.*

Received for publication July 6, 1954.

THE growth characteristics and morphology of transplanted mammary fibroadenomata and the effect of endogenous and exogenous hormones upon them have been presented in the two preceding papers (Millar and Noble, 1954*a* and 1954*b*). A transformation to fibrosarcoma occurred fairly frequently in such benign tumours and in many cases it was possible to detect fibrosarcomatous tissue within a fibroadenoma by macroscopic examination alone. By transplanting this tumour tissue many sub-lines of fibrosarcomata have been developed from the single line of fibroadenomata studied. The growth characteristics and pathology of these sub-lines are described briefly herein and compared with the fibroadenomata from which they originated.

There is considerable evidence in the literature to indicate that the fibrosarcomatous transformation constitutes a permanent modification of the growth characteristics of the original fibroadenoma. Increased takes (Heiman, 1934; Selbie, 1941) and growth-rate (Emge, 1938) have been noted when transplanted fibrosarcomata of fibroadenomatous origin were compared with the fibroadenoma. These tumours are reported to grow equally well in male and female rats (Oberling, Guerin and Guerin, 1935; Selbie, 1941) and in rats of different strains (Heiman 1934), characteristics not common to fibroadenomata. Heiman (1934) also found that the amount of X-radiation required to kill fibrosarcoma and fibroadenoma cells was 3500 r and 5500 r respectively. In order to produce further evidence of this alteration in response to environmental conditions, experiments were undertaken to determine the effect on transplanted fibrosarcomata of hormonal preparations known to influence the growth of the benign fibroadenoma. These were diethylstilbestrol at high dose levels and an anterior pituitary preparation. The former depressed while the latter enhanced fibroadenoma growth (Millar and Noble, 1954*b*).

### METHODS.

Fibrosarcoma lines developed from fibroadenoma were identified by the letters *FM.*— : a number before these letters distinguishing the individual lines and a number following, the transplant generation. At least one tumour of each transplant generation was examined microscopically.

Young adult female rats of the Sprague-Dawley strain were used in these studies. Details of transplantation technique (using single axillary implants) and the method for assessing the effects of different treatments within transplant series is given in the first of the two preceding papers (Millar and Noble, 1954*a*). For convenience a brief review of the latter is given below. After growth initiation, the tumours were measured once or twice weekly (depending on the growth-rate) till the death of the animal. Tumour size (in square inches) was plotted against time and from such growth curves figures were obtained for " latent " and " growth " periods. These were defined respectively as the time from the implantation date till the tumour was 0·4 square inches in area and the time for the tumour to grow from 0·4 to 3·0 square inches. Mean tumour latent and growth periods for treated and control groups were compared by the " t " test of Fisher, using a probability of 0·05 as the level of significance. All means will be presented with their standard errors.

Three separate experiments using different FM tumour lines were conducted to determine the effect of estrogen at high dose levels and a beef anterior pituitary suspension on the growth of the fibrosarcomata. Diethylstilbestrol was given orally as a constituent of the drinking water (0·5 mg. per cent) or by subcutaneous injection in sesame oil solution. Dose levels were approximately 50 $\mu$g. daily by the former and 200 $\mu$g. every second day by the latter mode of administration. As an added control in one experiment, a group of rats was subjected to dietary restriction sufficient to produce weight loss comparable to that observed in the estrogen injected rats. Preparation of the beef anterior pituitary saline suspension is outlined in a previous paper (Millar and Noble, 1954*b*). It was administered every second day by subcutaneous injection in an amount equivalent to 200 mg. of anterior pituitary substance (1 ml.). All treatment began at the time of tumour implantation and was continued throughout the duration of the experiment.

### RESULTS.

### (1) *Pathology.*

The first paper of this series (Millar and Noble, 1954*a*) gave a detailed description of the macroscopic and microscopic pictures of fibroadenomata which have undergone a fibrosarcomatous transformation. Variation in the degree of cellularily, anaplasia and mitotic activity led to the classification of this tissue as suspicious-low, low, moderate and high grade fibrosarcoma. Bony and cartilagenous metaplasia was observed in some tumours and has become quite characteristic of one FM tumour line after the second generation. Photomicrographs of a high grade fibrosarcoma and the metaplastic change were shown in the earlier paper (Millar and Noble, 1954*a*, Fig. 6 and Fig. 7).

Forty-two fibroadenomata suspected of having undergone a change to fibrosarcoma were transplanted. Of these, six grew as unchanged fibroadenomata, two were identified as fibroma, while the remainder fell into the above grades of fibrosarcomata. Although the bulk of the FM lines were classified initially as suspicious low-grade or low-grade fibrosarcomata, these tumours tended to change to the more cellular, anaplastic forms in the course of transplantation. In only one instance was there a reversion to a lower grade (37 FM to 37 FM1—moderate to low-grade fibrosarcoma).

### (2) *Growth characteristics of FM tumour lines.*

The growth energy of FM tumour lines varied considerably. The percentage takes ranged between zero and 100 ; the majority being 50 to 75 per cent in small groups of four or five rats. Latent periods for first generation tumours ranged from 7 to 61 days. Growth was usually fairly rapid, but as the tumours were measured only when experiments such as those to be reported were being conducted, quantitative data is not complete for this aspect. The mean " growth periods " given in Table I are representative of most of the tumours observed, although considerably slower growth than these figures indicate was not uncommon. Metastases have not been observed.

Correlation between the pathological grade of fibrosarcoma and the growth characteristics was by no means clear cut. With reference to the latent periods given above, only the lines originating from high-grade fibrosarcomata gave any indication of being a distinct group. The range for these tumours was 8 to 20 days, while the lower grades varied within the limits given, i.e., 7 to 61 days. In general it can be said only that the chances of obtaining rapidly growing, easily transplantable tumours, from tissue classified as moderate or high-grade fibrosarcoma exceed those from the two lower grades.

The pathological change from fibroadenoma to fibrosarcoma was accompanied by alterations in growth characteristics of the tumour and the response of the host to the tumour mass. Although there was considerable overlap in the range of latent and growth periods for fibroadenomata and fibrosarcomata, as a group of the FM tumours were more readily propagated and faster growing. The effect of the fibrosarcomata on the general health of the host varied considerably for individual tumours. Slow growing tumours were usually well tolerated, while the more rapidly growing ones were more deleterious to the health of the tumour-bearer. However, in no instance has an FM tumour reached a size of over 150 g. (5·0 to 7·0 square inches) without causing weight loss and cachexia. Death has occurred in some animals before the tumour has reached 3·0 square inches in size and could be attributed to no other obvious pathology. The depletion during the growth period of several groups may be noted in Table I. On the other hand, fibroadenomata of size equal to or exceeding the body-weight of the host do not appear to harm the tumour-bearer except through the physical disability resulting from the enormous tumour mass.

(3) *The effect of estrogen at high dose levels and an anterior pituitary preparation on the growth of FM tumours.*

The tumour lines used in these experiments were as follows :

15 FM2—: high-grade fibrosarcoma
35 FM5— : moderate-grade fibrosarcoma (35 FM4—: low-grade fibrosarcoma)
42 FM2— : high-grade fibrosarcoma.

The results for the experimental groups of each tumour line are given in Table I.

The mean latent period and the mean growth period of 15 FM and 35 FM lines respectively were significantly longer in diethylstilbestrol treated rats. A similar trend was noted in the 42 FM line, but the differences were not significant. Dietary restriction caused no retardation in tumour-growth in this experiment. The anterior pituitary suspension did not alter the growth trend of any of the three tumour lines studied.

TABLE I.—*The Effect of Estrogen at High Dose Levels and an Anterior Pituitary Suspension on the Growth of FM Tumours.*

| Tumour line. | Treatment. | No. of rats. | No. of takes. | Mean latent period (days). | Mean growth period (days). |
|---|---|---|---|---|---|
| 15 FM | Diethylstilbestrol* | 8 | 8 | 13±1·4‖ | 22±3·1 (5 rats) |
| | Anterior pituitary suspension | 8 | 8 | 9±0·3 | 18±2·5 |
| | Controls | 8 | 5 | 9±0·4 | 18±1·9 |
| 35 FM | Diethylstilbestrol† | 8 | 8 | 26±1·0 | 22±1·5‖ (6 rats) |
| | Anterior pituitary suspension | 8 | 6 | 25±0·6 | 13±1·3 (4 rats) |
| | Controls | 8 | 6 | 27±1·4 | 15±1·6 (5 rats) |
| 42 FM | Diethylstilbestrol† | 8 | 8 | 17±1·1 | 23±1·6 (7 rats) |
| | Anterior pituitary suspension | 8 | 8 | 15±1·5 | 18±1·1 |
| | Dietary restriction‡ | 8 | 8 | 13±0·4 | 19±1·9 |
| | Controls | 8 | 8 | 14±1·4 | 21±1·8 (7 rats) |

* Diethylstilbestrol given orally in drinking water—approximately 50 μg. daily.
† Diethylstilbestrol injected subcutaneously—200 μg. every second day.
‡ Dietary restriction—sufficient to produce weight loss, comparable to that observed in diethylstilbestrol injected rats.
‖ $P < 0·05$—Treated versus controls.

### DISCUSSION.

The results presented are in accordance with those reported by others. The sub-lines of fibrosarcomata arising from a single fibroadenoma line produced in general more rapidly growing, independent tumours than the fibroadenomata. The body weight loss and early death of fibrosarcoma-bearing rats was not observed for rats bearing fibroadenomata of similar or much larger size. Similarly, Emge (1938) reported that such fibrosarcomata caused the death of the host in 30 to 40 days. Metabolic differences have also been noted. Begg, Dickinson and Millar (1953) observed that the reduction in liver catalase activity was significantly greater in rats bearing transplanted fibrosarcomata or fibroadenomata of which a portion had become sarcomatous than in rats bearing totally benign fibroadenomata.

The histological appearance and the growth properties point to the classification of these tumours as malignant growths. Metastases have not been observed in FM tumour-bearing rats, but Emge (1938) noted that these fibrosarcomata recurred rapidly after surgical removal, while Heiman (1934) observed local invasion into the chest wall of one such tumour. Increased autonomy as shown by diminished or negative response to hormonal factors known to influence the fibroadenoma offers further evidence of the true malignancy of these growths. Diethylstilbestrol administered at similar dose levels prevented the initiation of growth in fibroadenoma implants, or arrested or markedly slowed the growth of growing tumours (Millar and Noble 1954*b*). The comparatively slight, but usually significant growth retardation shown by FM tumours was similar to that observed with estrogen administration for other malignant growths, including sarcomata (Nathanson and Salter, 1939; Eisen, 1941). Fibroadenoma-stimulating hormones present in beef anterior pituitaries did not enhance the growth of FM tumours. This confirms the alternate findings of Emge and Murphy (1936)

that growth of similar tumours in hypophysectomised rats was only slightly depressed, if at all.

The existence and propagation of benign and malignant growths of common origin offers a tool for cancer research which may be of considerable value. The use of the benign tumour-bearer as a more representative control for the study of malignant tumour-host relationships has already been reported for liver-catalase studies by Begg, Dickinson and Millar (1953). It is possible too that the more gradual development of malignancy from normal tissue through the benign neoplasm, would reveal in metabolic studies more subtle changes than can be detected in comparing only normal and malignant tissue of common origin.

### SUMMARY.

1. The pathology and growth characteristics of tumour lines originating from fibrosarcomatous tissue occurring in transplanted mammary fibroadenomata are described.

2. In comparing these tumours with the benign growths of their origin, it was noted that the fibrosarcomata were usually faster growing and more easily propagated than the fibroadenomata. In addition they exerted a deleterious effect upon the health of the host, causing weight loss and death. No comparable response was observed in rats bearing fibroadenomata.

3. Diethylstilbestrol at high dose levels caused slight but usually significant retardation of tumour growth, a response similar but not comparable in degree to that observed with the fibroadenoma.

4. A crude beef anterior pituitary suspension known to enhance the growth of fibroadenomata did not alter the growth energy of fibrosarcomata.

This work has received continuous financial support from the National Cancer Institute of Canada. The authors are grateful for the assistance of Dr. J. C. Paterson in preparing and evaluating the microscopic sections.

Miss Bette Byrns rendered valuable technical assistance in these experiments.

### REFERENCES.

BEGG, R. W., DICKINSON, T. E., AND MILLAR, J.—(1953) *Canad. J. med. Sci.*, **31**, 315.
EISEN, M. J.—(1941) *Cancer Res.*, **1**, 457.
EMGE, L. A.—(1938) *Arch. Path.*, **26**, 429.
*Idem* AND MURPHY, K. M.—(1936) *Amer. J. Obstet. Gynec.*, **32**, 593.
HEIMAN, J.—(1934) *Amer. J. Cancer*, **22**, 497.
MILLAR, M. J., AND NOBLE, R. L.—(1954*a*) *Brit. J. Cancer*, **8**, 485.—(1954*b*) *Ibid.*, **8**, 495.
NATHANSON, I. T., AND SALTER, W. T.—(1939) *Arch. Path.*, **27**, 828.
OBERLING, C., GUERIN, M., AND GUERIN, P.—(1935) *Bull. Ass. franc. Cancer*, **24**, 232.
SELBIE, F. R.—(1941) *Brit. J. exp. Path.*, **22**, 156.